# Acute and Preventive Management of Migraine during Menstruation and Menopause

**DOI:** 10.3390/jcm10112263

**Published:** 2021-05-24

**Authors:** Raffaele Ornello, Eleonora De Matteis, Chiara Di Felice, Valeria Caponnetto, Francesca Pistoia, Simona Sacco

**Affiliations:** Neuroscience Section, Department of Applied Clinical Sciences and Biotechnology, University of L’Aquila, 67100 L’Aquila, Italy; raffaele.ornello@univaq.it (R.O.); eleonoradematteis@fastwebnet.it (E.D.M.); chiaradi.felice@yahoo.com (C.D.F.); valeria.caponnetto@univaq.it (V.C.); francesca.pistoia@univaq.it (F.P.)

**Keywords:** migraine, menstruation, menopause, migraine treatment, triptans, hormonal treatments

## Abstract

Migraine course is influenced by female reproductive milestones, including menstruation and perimenopause; menstrual migraine (MM) represents a distinct clinical entity. Increased susceptibility to migraine during menstruation and in perimenopause is probably due to fluctuations in estrogen levels. The present review provides suggestions for the treatment of MM and perimenopausal migraine. MM is characterized by long, severe, and poorly treatable headaches, for which the use of long-acting triptans and/or combined treatment with triptans and common analgesics is advisable. Short-term prophylaxis with triptans and/or estrogen treatment is another viable option in women with regular menstrual cycles or treated with combined hormonal contraceptives; conventional prevention may also be considered depending on the attack-related disability and the presence of attacks unrelated to menstruation. In women with perimenopausal migraine, hormonal treatments should aim at avoiding estrogen fluctuations. Future research on migraine treatments will benefit from the ascertainment of the interplay between female sex hormones and the mechanisms of migraine pathogenesis, including the calcitonin gene-related peptide pathway.

## 1. Introduction

Migraine is a common headache disorder affecting 14% of people worldwide [1]. Migraine commonly affects young people and especially women; indeed, the prevalence of migraine is three to four times higher in women compared with men [1,2,3]. Different mechanisms might explain the female predominance of migraine, including the migraine-enhancing role of female sex hormones and a higher genetic heritability in women compared with men [4]. Of note, migraine prevalence is the same in women and in men during childhood, while a higher prevalence of migraine in women than in men is found only after puberty, when women start menstruating [5]. According to the so-called “estrogen withdrawal hypothesis”, the cycles of rise and fall in estrogen levels typical of the female fertile period are deemed responsible for an increased susceptibility to migraine in women [6]. Estrogen levels undergo a sudden fall during the days immediately preceding menstruation [7,8]. Hence, the highest susceptibility to migraine is in the perimenstrual period. The years immediately preceding and following the menopause also pose women at an increased risk of having severe migraine [9] due to the rapid fluctuations in estrogen levels that accompany this phase of the female reproductive cycle.

**Figure 1:**
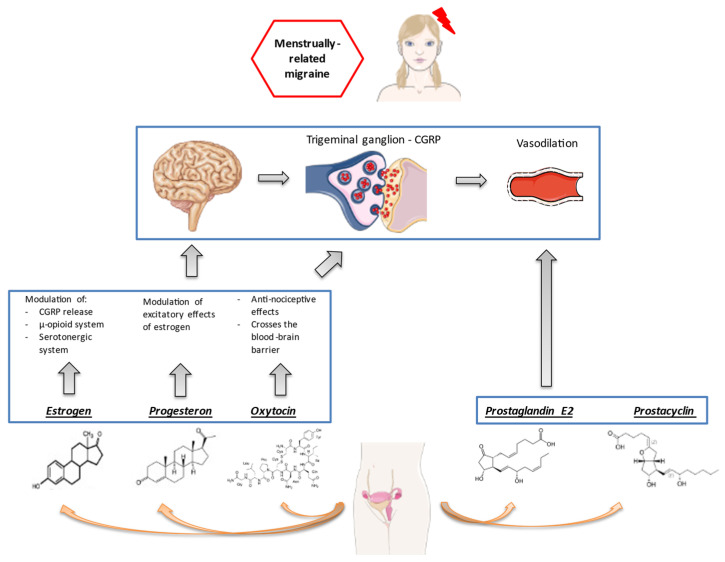
Female sex hormones and pathogenesis of menstrual migraine.

Estrogen withdrawal can partly explain the effect of female sex hormones on migraine; however, the influence of sex hormones on migraine mechanisms is complex and not fully elucidated. Sex hormones influence nociceptive processing, acting on both central and peripheral pathways [10]. Animal studies have found a broad localization of estrogen receptors in brain structures involved in migraine generation, including the trigeminal ganglion [11] and pain-processing structures within the brain [12]. At a central level, estrogens can activate the endogenous opioid system that controls pain sensations [13] and modulate the levels of oxytocin, a neurohormone released by the hypothalamus, which can exert an anti-migraine effect [14]. Notably, both estrogens [15] and oxytocin [16] might act on the trigeminal ganglion and modulate the release of calcitonin gene-related peptide (CGRP), which is implied in the generation of migraine pain. Progestins might also attenuate pain responses via conversion to the neurosteroid allopregnanolone, as suggested by animal evidence [17]. Taken all together, female sex hormones influence the susceptibility to migraine, acting through a complex interplay of central and peripheral pathways (Figure 1).

**Figure 2:**
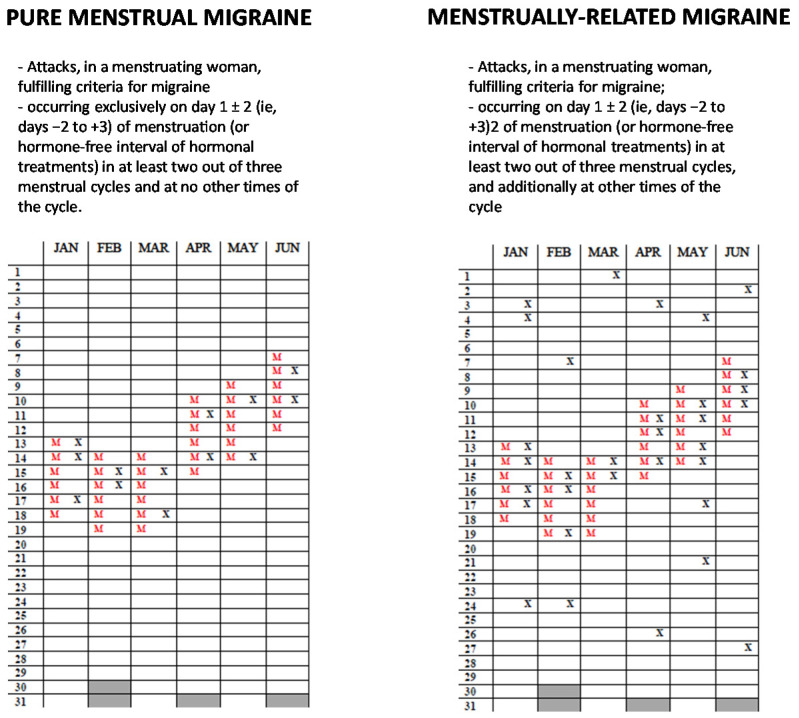
Example of headache diary of patient with pure menstrual and menstrual-related migraine. The Xs represent headache days; Ms represent reported menstruation days.

The influence of female hormonal fluctuations on migraine is so remarkable that menstrual migraine (MM) is recognized by the current international classification as a distinct headache disorder [18]. In detail, pure menstrual migraine occurs exclusively on day 1 ± 2 of menstruation in at least two out of three menstrual cycles and at no other times of the cycle, while menstrual-related migraine occurs on day 1 ± 2 of menstruation in at least two out of three menstrual cycles, and additionally at other times of the cycle [18] (Figure 2). Differently from MM, perimenopausal migraine is not recognized as an independent clinical entity.

The treatment of MM and perimenopausal migraine generally follows the indications for the acute and preventive treatment of any other form of migraine, in the absence of specific indications. Nevertheless, some additional considerations are needed when migraine occurrence is influenced by female sex hormones.

In the present paper, we narratively reviewed the current concepts on the treatment of menstrual and perimenopausal migraine and the possible lines of future treatments, with a focus on practical suggestions.

## 2. Methods

We searched PubMed and Scopus for articles containing the terms “migraine”, “menstrual”, “menopause”, and “treatment”, up to February 2021. We included reviews and original articles. Due to the broadness of the topic and the inclusion of the authors’ personal views, we did not perform a systematic review.

## 3. The Burden and Unmet Needs of Women with Menstrual and Perimenopausal Migraine

MM affects 3% of young women [19], with a peak of 22% in women aged 30–34 years [20]. According to several studies, migraine attacks occurring during the perimenstrual period are longer, more severe, have more associated symptoms, and are less responsive to acute medication compared with non-menstrual migraine attacks [21,22,23,24]. Menstruation is a poorly controllable hormonal trigger which exposes women to anticipatory anxiety of headaches, so-called “cephalalgiaphobia” [25]; this fear of headache could lead, in turn, to increased consumption of painkillers [26].

The reported prevalence of migraine during the menopause is 10–29% [9]. The years immediately preceding and following the menopause are characterized by an increased susceptibility to migraine [27,28] due to the frequent rise and fall of estrogen levels before reaching stable low levels years after the menopause [9]. After natural menopause, migraine usually improves [28,29], while women undergoing surgical menopause have a high risk of migraine worsening, possibly due to a sudden fall in estrogen levels [30,31]. The proportion of women reporting postmenopausal migraine worsening is particularly high in headache clinics, possibly due to selection bias [9,32]. Therefore, headache clinics likely provide care to a high proportion of women with perimenopausal migraine worsening, and women with previously rare migraines can come to the attention of a headache center for the first time because of a perimenopausal worsening.

**Figure 3:**
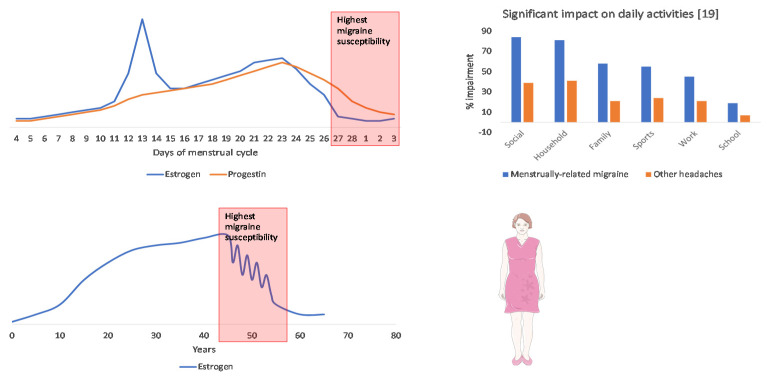
Graphical representation of the menstrual and perimenopausal periods of increased susceptibility to migraine and women’s quality of life impairment. The panels on the left indicate the levels of female sex hormones during the menstrual cycle (above) and the years of women’s life (below). The graph on the right shows the proportion of women reporting impairment of daily activities due to menstrual-related migraine (blue columns) compared with other headaches (orange columns; data from [19]).

The high burden of hormonally triggered migraine in women can lead to decreased quality of life and high disability (Figure 3). However, specific strategies for the management of hormonal triggers need to be better defined. 

## 4. Acute Treatment of Menstrual Migraine

### 4.1. Triptans

There is currently no acute treatment specifically designed for MM. The use of triptans for the treatment of MM has been extensively evaluated. Randomized controlled trials and open-label studies have established the efficacy of sumatriptan [33,34,35], frovatriptan [36], naratriptan [37], zolmitriptan [38], and almotriptan [39] for the acute treatment of MM (Table 1). All triptans were more effective than placebo in relieving pain during the first 2–4 h; however, most triptans were not superior over placebo in preventing migraine recurrence within 24 h from taking the drug [33,34]. Frovatriptan had better performances over the other triptans [40,41,42], possibly due to its longer half-life; hence, it could be advisable for use in MM, whose attacks are usually longer compared with those in the intermenstrual period [4].

### 4.2. Common Analgesics

Although being migraine-specific, triptans are not the only drugs that can be used for the acute treatment of MM. The use of simple analgesics in MM is justified by the increased release of prostaglandins during the perimenstrual period, which leads not only to perimenstrual pain, but also to an increased proneness to migraine. This hypothesis is supported by the increase in prostaglandin salivary levels which was found in women with migraine and associated dysmenorrhea [43]; further studies found an association between migraine and dysmenorrhea [44] and that the infusion of prostaglandins can induce migraine attacks in women with migraine with aura [45]. Mefenamic acid, an inhibitor of prostaglandin synthesis, effectively treated MM in an early trial [46]. The commonly used nonsteroidal anti-inflammatory drugs inhibit prostaglandin synthesis by inhibiting cyclooxygenase enzymes [47] and can therefore be used in the acute treatment of MM, especially in women with dysmenorrhea. Except from mefenamic acid, there are no studies of nonsteroidal anti-inflammatory agents specifically addressing their use in MM.

### 4.3. General Considerations on the Acute Treatment of MM

It is important that drugs are taken soon after the beginning of pain, when it is still mild, to maximize their efficacy [35]. This treatment principle is far more important for menstrual episodes, which impose a high burden of pain on women compared with non-menstrual episodes. Not only the type of drugs, but also their mode of administration can influence their effectiveness in the acute management of MM; as MM is associated with an increased occurrence of gastrointestinal symptoms, such as nausea and vomiting, using non-oral formulations can contribute to increasing the effectiveness of acute treatments. This view is supported by a pilot trial which showed a higher performance of sumatriptan suppositories over sumatriptan tablets in relieving pain, associated symptoms, and pain recurrence [48].

As stated before, MM attacks tend to require a high use of acute treatment. Triptans and common analgesics act on different pathways; therefore, combining triptans with analgesics is an alternative approach to effectively treat MM. The available trials confirmed the efficacy of sumatriptan plus naproxen [49,50,51,52] and of frovatriptan plus dexketoprofen [53] (Table 1). Common analgesics can also be used as a rescue treatment if triptans fail. It is advisable that patients adopting rescue treatments take a triptan first to maximize its efficacy; common analgesics can be taken soon after the triptan, maintaining gastrointestinal safety.

**Table 1:** Summary of drugs for the acute treatment of menstrual migraine. OLS indicates open-label study; RCT, randomized controlled trial.

Drug	Available Studies	Main Findings
*Single drug*		
Frovatriptan	5 RCTs vs. other triptans [40,41,42]; 1 OLS	Early relief: superior to placebo, equivalent to other triptans Sustained relief: superior to almotriptan, rizatriptan, zolmitriptan Non-headache symptoms: effective on nausea and phonophobia, not on other symptoms Adverse events: comparable to placebo Other outcomes: higher patient satisfaction with frovatriptan compared with previous treatments
Sumatriptan	3 RCTs [33,34,35]	Early relief: superior to placebo Sustained relief: comparable to placebo Non-headache symptoms: effective on photophobia and phonophobia Adverse events: comparable to placebo
Naratriptan	1 RCT [37]	Early relief: superior to placebo Sustained relief: superior to placebo Non-headache symptoms: superior to placebo for all symptoms Adverse events: comparable to placebo Other outcomes: superior to placebo in ability to carry on daily activities and patient satisfaction
Zolmitriptan	1 RCT [38]	Early relief: superior to placebo Adverse events: comparable to placebo
Almotriptan	1 RCT [39]	Superior to placebo in pain-free status at 2 and 24 h; significant reduction in nausea and photophobia; adverse events comparable to placebo
*Combination drugs*		
Sumatriptan + naproxen	5 RCTs [50,51,52]	Early relief: superior to placebo Sustained relief: superior to placebo, especially with comorbid dysmenorrhea Adverse events: comparable to placebo Other outcomes: patient satisfaction, productivity, quality of life
Frovatriptan + dexketoprofen	1 RCT [53]	Early relief: superior to frovatriptan alone Sustained relief: superior to frovatriptan alone Adverse events: comparable to frovatriptan alone

When considering the management of MM, as well as of migraine in general, the treating physician should consider the woman’s comorbidities that might influence the hormonal status. Collaboration between headache physicians and gynecologists is encouraged to manage menstrual irregularities and reproductive conditions that can influence migraine. Notably, some medical comorbidities, such as obesity, have a higher impact on women than on men [54,55,56]. Adipokines and leptin have been implied in the interaction between obesity and migraine [57], and obesity has a complex relationship with female sex hormones in terms of appetite regulation and adipose tissue distribution [58]. The management of MM should take into account the whole medical condition of women and all the possible internal and external influencing factors.

## 5. Prevention of Menstrual Migraine

The available migraine preventive agents are also effective on MM, as shown by an open-label study on topiramate [59] and by a post hoc analysis of a randomized controlled trial of erenumab [60]. However, migraine tends to have a menstrual-related pattern even under effective prevention, with more frequent episodes in the perimenstrual than in the non-menstrual period, as shown by open-label studies on topiramate and erenumab [59,61]. This means that conventional preventatives cannot counterbalance the hormonal trigger. For this reason, specific hormonal strategies may be needed if a woman continues to consistently experience disabling menstrual attacks despite effective ongoing prevention for non-menstrual attacks. 

Preventive treatment is started when migraine significantly interferes with the patient’s life [62]. In the case of MM, and mostly pure menstrual migraine, the impairment in quality of life can be severe, although only during a few days each month. Hence, common migraine preventatives, which are prescribed for daily use, might not have a net benefit. It is useful to plan MM-specific strategies for prevention, especially in women with pure MM. Such strategies include short-term prevention and hormonal manipulation.

### 5.1. Short-Term Prevention with Triptans or NSAIDs

Short-term prevention of MM is particularly useful in women taking oral contraceptives or with very regular menstrual cycles, in whom the period of maximum susceptibility to migraine is easily predictable. It can also be applied as an integration to standard prophylaxis in women with menstrual-related migraine not adequately controlled by standard prevention. This kind of treatment focuses on decreasing the burden of migraine during the days of highest susceptibility, while avoiding continuous prophylaxis and its potential adverse events. When considering a short-term prophylaxis, it should be kept in mind that natural menstrual cycles are subject to huge variations; according to a study, the perimenstrual period can be accurately predicted only in 27% of cases [63]. The available trials have assessed the efficacy of common analgesics, including naproxen [64] and nimesulide [65], and several triptans, including frovatriptan [66,67,68], naratriptan [69,70], and zolmitriptan [71] [Table 2]; all those drugs were taken for 5 to 14 days around the start of menstruation. The trials’ results are largely positive, with the highest quality of evidence available for frovatriptan and naratriptan [4]. Higher doses of triptans were generally more effective than lower doses, while maintaining safety (Table 2). However, the daily use of triptans for the short-term prevention of MM has the drawback of possible ineffectiveness in women with irregular duration of menstrual cycles. Besides, the daily use of triptans can expose women to medication overuse [72]. It might be advisable to prescribe triptans as short-term prevention only to women with very regular menstrual cycles or under combined hormonal treatments and pure MM to maximize efficacy while minimizing the risk of medication overuse.

### 5.2. Hormonal Prevention

Hormonal manipulation strategies are an alternative option for the management of MM based on the estrogen withdrawal hypothesis. These strategies aim at preventing MM by reducing or suppressing the fall in estrogen levels that precedes menstruation. This goal can be achieved by short-term hormonal supplementation or by the continuous use of exogenous hormones in fixed doses, without hormone-free intervals. It should be kept in mind that the intermittent formulations of oral combined hormonal contraceptives usually exacerbate migraine as they lead to higher fluctuations in estrogen levels compared with natural menstrual cycles [73,74].

As far as short-term estrogen supplementation is concerned, several trials have assessed the efficacy of estrogen gel [75,76,77] or patches [78,79] to mitigate the estrogen fall during the perimenstrual days (Table 2). Following a similar strategy, a further open-label study suggested the efficacy of an oral contraceptive containing 20 μg ethinyl estradiol on days 1 to 21, supplemented with 0.9 mg conjugated equine estrogens on days 22 to 28, for the prevention of MM [74]. It is important to note that short-term estrogen supplementation is effective as long as it counteracts estrogen withdrawal; hence, the best use of estrogen supplementation is during the whole estrogen-free interval of oral contraceptives, especially if containing low estrogen doses [74].

**Table 2:** Summary of drugs for the short-term prophylaxis of menstrual migraine. RCT indicates randomized controlled trial.

Drug	Available Studies	Treatment Protocol	Main Findings
*NSAIDs*			
Naproxen	1 RCT [64]	500 mg twice daily for 14 days for 3 cycles	Significant reduction in number, duration, and severity of attacks compared with placebo only during the 2nd and 3rd cycle
Nimesulide	1 RCT [65]	100 mg thrice daily for 10 days for 3 cycles	Significant reduction in pain intensity and duration compared with placebo during all the cycles
*Triptans*			
Frovatriptan	2 RCTs, 1 open-label extension [66,67,68]	2.5 mg daily or twice daily for 6 days	Significant reduction in headache days, headache intensity, headache duration, and use of rescue medication; twice daily formulation better than daily formulation
Naratriptan	1 RCT [70]	1 mg or 2.5 mg twice daily for 5 days for 3 cycles	Significant reduction in headache days, headache intensity, headache duration, and use of rescue medication; significant improvement in quality of life; 2.5 mg dose better than 1 mg dose
Zolmitriptan	1 RCT [71]	2.5 mg twice or thrice daily for 7 days for 3 cycles	Significant reduction in headache days, pain recurrence, and rescue medication with both doses
*Hormone supplementation*			
Percutaneous estradiol	3 RCTs [75,76,77]	7–10 days for 2–3 cycles	Significant reduction in headache days and acute medication use, only during the treatment, with subsequent rebound headache; good tolerability profile
Transdermal 17 β-estradiol	1 RCTs [78]	6 days for 3 cycles	Estradiol effective only if synchronized with menstruation
Conjugated equine estrogens	1 Open-label [74]	7 days (hormone-free interval of a combined contraceptive) for 2 cycles	At least 50% reduction in monthly headache days in all treated women; improvement in menstrual symptoms

**Figure 4:**
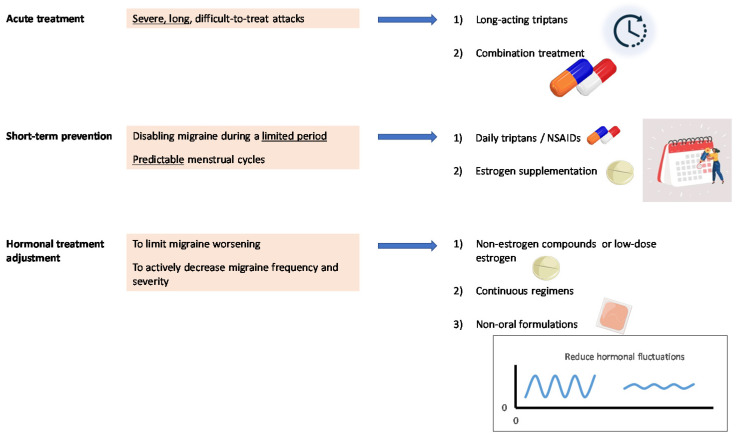
Suggestions for the treatment of menstrual migraine in clinical practice.

An alternative strategy to prevent MM in women seeking contraception is to use specific formulations of low-dose estrogens and/or progestins, such as estradiol valerate plus dienogest [80]. Continuous administration of estrogen, without [81] or with a reduced time hormone-free interval [82], or the use of non-oral formulations, such as the vaginal ring [83], also showed efficacy in decreasing the burden of MM (Figure 4). 

An alternative modality of hormonal manipulation is the use of phytoestrogen; the daily use of a combination of soy isoflavones, dong quai, and black cohosh, all of which contain phytoestrogens, effectively decreased headache frequency in women with menstrual-related migraine without aura in a randomized controlled trial [84]. A further trial suggested the efficacy of the soy isoflavones genisteine and diadzeine in controlling MM [85]. Using phytoestrogens instead of the usual estrogen formulations could also mitigate the vascular risk associated with combined hormonal contraception [86], which is an important concern in women with migraine [87]. 

Hormonal treatments differentially affect migraine with and without aura, as migraine without aura tends to follow a menstrual-related pattern more frequently than migraine with aura [88,89]. Female sex hormones affect the two forms of migraine in different ways; while estrogen withdrawal is a trigger for migraine without aura, high estrogen states are a trigger for migraine aura [89]. This clinical finding is supported by animal models in which high estrogen states acted as a trigger for cortical spreading depression, the pathophysiological correlate of aura [90]. It is likely that women with migraine with aura undergo migraine exacerbations if exposed to combined hormonal contraceptives and mostly those with high estrogen doses. Besides, women with migraine with aura have a highly increased vascular risk, which could be further increased by using combined hormonal contraceptives [91]. Therefore, the use of exogenous estrogens should be best avoided in women reporting auras.

The induction of pharmacological menopause could theoretically prevent MM by stopping the cycles of continuous rise and fall of estrogen levels that exacerbate migraine; however, evidence suggests that medically induced menopause alone is not adequate for migraine prevention, and that adding exogenous estradiol provides only modest preventive benefit [92]. 

### 5.3. Non-Pharmacological Treatments

Non-pharmacological treatments can be useful in women with MM who refuse or have contraindications to hormonal or other pharmacological treatments; in addition, they can be used as adjunctive short-term treatments. A small trial showed the efficacy of 360 mg daily magnesium in decreasing MM frequency compared with placebo [93]. Another small randomized controlled trial showed the efficacy of 400 daily units of vitamin E over placebo in decreasing pain intensity and headache-related disability [94]. Notably, some nutrients, and mostly magnesium, are also useful for the treatment of premenstrual syndrome [95]. Herbal products have limited but promising evidence in the treatment of MM overlapping with premenstrual symptoms; vitex agnus-castus showed effective control of those symptoms in an open-label study [96]. A randomized controlled trial showed the benefit of transcranial direct current stimulation in MM over placebo [97]; however, this technique is of limited availability. From a practical point of view, we deem it reasonable to prescribe perimenstrual nutrients such as magnesium to women reporting disabling pure MM associated with premenstrual symptoms.

## 6. Considerations on the Treatment of Perimenopausal Migraine

The menopausal transition is a process occurring over several years characterized by wide fluctuations that ends in stable low levels of both estrogens and progestins [98]. The susceptibility to migraine is highest in the late premenopausal and perimenopausal period, when hormonal fluctuations reach their maximum [9]. The hormonal fluctuations of perimenopause are associated with the occurrence of mood changes, weight gain, and vasomotor symptoms such as hot flashes, which are particularly common in women with migraine [99]. There are no specific guidelines for the preventive treatment of migraine during the menopausal transition; nevertheless, some special considerations may apply to that population of women regarding both hormonal and non-hormonal treatments.

### Hormonal Treatments

Hormonal replacement therapy (HRT). HRT is usually prescribed to reduce the vasomotor symptoms associated with the menopausal transition [100,101]. However, population-based studies suggest that HRT worsens migraine [32,102,103]. To limit migraine worsening after the menopause, different strategies are viable based on the estrogen withdrawal hypothesis, including the continuous rather than intermittent administration of combined hormonal treatments [104], the intrauterine or transdermal rather than oral administration of estrogen [105], and the administration of non-estrogen compounds such as tibolone [106]. An alternative strategy could be the use of phytoestrogens such as soy isoflavones, which can control menopausal symptoms [107] together with hormone-related migraine. The types and doses of estrogens and progestins in HRT can also have a different impact on migraine. As high-estrogen states can increase the susceptibility to migraine with aura [89], the use of the lowest possible doses of estrogens can contribute to avoiding the exacerbation of migraine during the menopausal transition. This result is suggested by an early case series of four patients developing migraine aura after starting HRT and in whom reducing the estrogen dose or avoiding the oral route of administration led to the disappearance of aura [108]. Referring to progestins, the use of products without androgenic properties or natural progesterone [109] or non-oral routes of administration [110] might be beneficial to migraine [111].

When considering hormonal treatments for postmenopausal women, the possible benefits should be compared with the risk of cerebrovascular events, which is increased both by migraine itself and by estrogens [91,112,113], even at the lowest doses [87]. Women with migraine, and mostly those with migraine with aura, who enter the perimenopausal period should be advised to avoid HRT if possible due to the potential harms to their health. Antidepressants such as escitalopram or venlafaxine can be used to control both vasomotor symptoms and migraine in postmenopausal women in whom HRT is contraindicated [114,115].

The efficacy of venlafaxine against perimenopausal vasomotor symptoms was comparable to that of HRT with estrogen according to a randomized clinical trial [116]; venlafaxine is also an effective treatment for migraine [117] and can therefore represent a valid alternative to hormonal treatments in the management of perimenopausal migraine. More limited evidence is available on the migraine-preventing effects of paroxetine [118], escitalopram [119], and gabapentin [120]; however, all those agents can also be used to treat hot flashes [121,122,123] and can therefore be effectively used for the treatment of migraine in perimenopause. Among non-pharmacologic treatments, vitamin E, acupuncture, and physical exercise have modest evidence of effect on both vasomotor symptoms and migraine [124].

**Figure 5:**
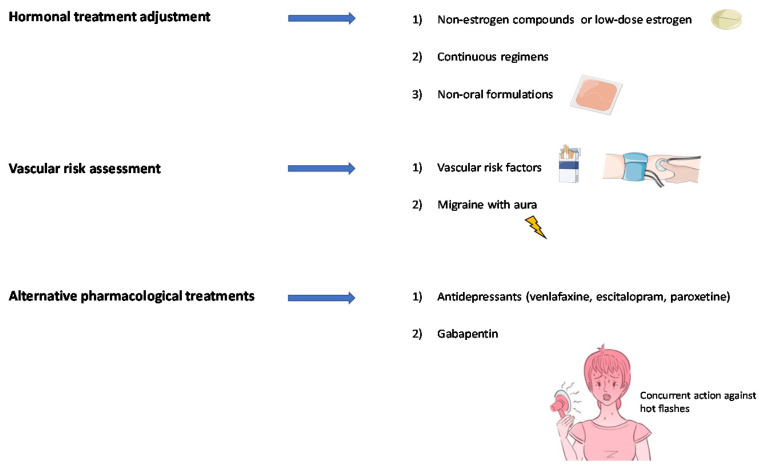
Suggestions for the treatment of perimenopausal migraine in clinical practice.

Figure 5 summarizes the suggestions for migraine treatment that may specifically apply to perimenopausal women.

## 7. Conclusions and Future Directions

The influence of female sex hormone fluctuations on migraine is well known. However, the mechanisms underlying that influence are still unclear, hindering the development of new treatments specific for hormone-related migraine. To date, the most specific treatments for menstrual and perimenopausal migraine are based on hormone manipulation, with the goal of limiting the fluctuations in estrogen levels that exacerbate migraine frequency and severity. Further studies are needed to understand the complex interplay between female sex hormones and the mechanisms of migraine generation. There is evidence that estrogens can modulate several neurotransmitter systems, including the serotonergic, GABAergic, and opioid systems in the brain [4]. High estrogen levels also increase the levels of calcitonin gene-related peptide (CGRP), which is strictly implied in the generation of migraine pain [10]. Given the high prevalence of migraine in women, the design of future migraine treatments should consider the interactions between sex hormones and migraine.

The current approach to migraine treatment includes acute treatments, which are given for each headache episode, and preventive treatments, which are given on a continuative basis for months to years. The so-called “short-term prophylaxis” of MM is in fact the continuous use of treatments designed to be acute, with the consequent risk of medication overuse, especially in women that cannot predict the duration of their menstrual cycles. There is a need for a more versatile approach to the treatment of MM that can overcome the distinction between acute and preventative treatments. Interestingly, gepants, the oral CGRP receptor antagonists, can act both as acute and as preventative migraine treatments [125]. This is particularly true for rimegepant, which has been proven effective in both roles by randomized controlled trials [126,127,128]. Telcagepant, taken perimenstrually for seven days, effectively reduced perimenstrual headache days, although not the absolute number of headache days, over placebo in a randomized controlled trial [129]. The clinical development of telcagepant was stopped because of tolerability issues; nevertheless, it suggests that gepants can be effective over migraine attacks exacerbated by female sex hormone fluctuations. Specific studies are needed for the specific 5-HT-1F receptors, ditans. Lasmiditan has been proven effective in two large randomized controlled trials [130,131] and we cannot exclude its efficacy on MM as it shares some mechanisms of action with triptans. Together with the development of new migraine treatments, combined hormonal treatment strategies should be refined to find the best combinations and ways of administration in women with MM and perimenopausal migraine, in order to achieve better migraine control or avoid migraine worsening.

As shown by the successful application of treatments acting on the CGRP pathway, elucidating the mechanisms of migraine can lead to significant improvements in its management. Uncovering the mechanisms by which female sex hormones influence migraine will likely lead to further improvements in the treatment of hormone-dependent headaches.
